# Structure of UBE2Z Enzyme Provides Functional Insight into Specificity in the FAT10 Protein Conjugation Machinery*

**DOI:** 10.1074/jbc.M115.671545

**Published:** 2015-11-10

**Authors:** Julien Schelpe, Didier Monté, Frédérique Dewitte, Titia K. Sixma, Prakash Rucktooa

**Affiliations:** From the ‡ UMR8576 CNRS-Université de Lille, 50 Avenue de Halley, 59658 Villeneuve d'Ascq, France and; § Division of Biochemistry and Centre for Biomedical Genetics, Netherlands Cancer Institute, Plesmanlaan 121, 1066 CX Amsterdam, The Netherlands

**Keywords:** crystal structure, post-translational modification (PTM), ubiquitin, ubiquitin-conjugating enzyme (E2 enzyme), ubiquitylation (ubiquitination), FAT10, UBE2Z, UBL conjugation

## Abstract

FAT10 conjugation, a post-translational modification analogous to ubiquitination, specifically requires UBA6 and UBE2Z as its activating (E1) and conjugating (E2) enzymes. Interestingly, these enzymes can also function in ubiquitination. We have determined the crystal structure of UBE2Z and report how the different domains of this E2 enzyme are organized. We further combine our structural data with mutational analyses to understand how specificity is achieved in the FAT10 conjugation pathway. We show that specificity toward UBA6 and UBE2Z lies within the C-terminal CYCI tetrapeptide in FAT10. We also demonstrate that this motif slows down transfer rates for FAT10 from UBA6 onto UBE2Z.

## Introduction

Fatylation is a post-translational modification yielding a covalent isopeptide bond between a ubiquitin-like modifier (UBL)^2^ called FAT10 and a target protein (1, 2). Proteins in the UBL family adopt a β-grasp fold and include ubiquitin NEDD8 or SUMO, but also ISG15, which is composed of two ubiquitin-like modules (3). FAT10 is an 18.4-kDa UBL present only in mammals. It comprises two consecutive ubiquitin-like domains (28 and 33% identity to ubiquitin, respectively, 18% to each other).

FAT10 is the only UBL other than ubiquitin that promotes proteasomal degradation once tethered onto target proteins (4, 5). It is constitutively highly expressed in mature dendritic cells as well as organs of the immune system (6). Its expression is also induced in almost all cell types by interferon-γ or TNF-α, pointing toward a role of FAT10 in the immune response (7). Recent work also shows that proteins involved in cell cycle regulation are differentially fatylated depending on cell cycle progression and indicate that FAT10 conjugation could be involved in regulating mitotic progression (8). Interestingly, FAT10 knock-out mice display phenotypic evidence of reduced adiposity, delayed aging, and extended lifespan, suggesting that this UBL acts as an immune metabolic modulator (9). Further studies correlate FAT10 overexpression with tumorigenesis (10–12) and suggest that FAT10 interaction with MAD2 is a key element in the pro-malignant property of this UBL (13).

FAT10 is conjugated onto target proteins in the same way as other UBLs, through a three-step enzymatic process. The UBL is first activated by a ubiquitin activating enzyme (E1) in an ATP-dependent manner, resulting in the covalent attachment of the carboxyl group of the UBL C-terminal glycine residue onto the E1 catalytic cysteine via a thioester bond. The activated UBL is further linked onto the catalytic cysteine of a ubiquitin conjugating enzyme (UBC or E2) through a trans-thioesterification reaction. The UBL is then generally transferred onto the ϵ-amino group of a lysine on the target protein, resulting in the formation of an isopeptide bond. This last step of the conjugation reaction can be catalyzed by an E3 ligase. The E1 and E2 enzymes associated with fatylation are UBA6 (also known as UBE1L2) (14, 15) and UBE2Z (also known as USE1) (15, 16), respectively, but so far no E3 ligase has been associated with FAT10 conjugation.

UBA6, the only E1 responsible for FAT10 activation, displays unusual dual substrate specificity toward both ubiquitin and FAT10 (14, 15). UBA6 shares ∼40% sequence identity with UBA1, the prototypical E1 enzyme involved in ubiquitin activation (15, 17), and displays selectivity in its ability to function with different E2 enzymes. Contrary to UBA1, UBA6 cannot transfer activated ubiquitin onto UBE2R1 or UBE2R2 (also known as CDC34B or CDC34A, respectively), E2 enzymes involved in cell cycle regulation (15). On the other hand, UBA6 but not UBA1 is capable of loading UBE2Z with ubiquitin. It has been shown that a UBA6 chimera containing the UBA1 C-terminal ubiquitin fold domain (UFD), involved in E2 recognition, can load ubiquitin on UBE2R1 or UBE2R2 but that a UBA1 chimera containing the UBA6 UFD cannot charge UBE2Z with ubiquitin (15). This suggests that different recognition mechanisms are involved, possibly similar to that between the SUMO E1 Cys domain and the SUMO E2 enzyme UBE2I (also known as UBC9) (18) or the NEDD8 E2 UBE2M (also known as UBC12) that uses an ∼40-residue-long N-terminal extension to fit into a groove found only in the NEDD8 E1 component UBA3 (19).

UBE2Z functions as an E2 enzyme downstream of UBA6 in the conjugation of either ubiquitin or FAT10 onto target proteins (15, 16). It is one of the ∼35 different ubiquitin conjugating enzymes described so far in humans (20). E2 enzymes (21) are globular proteins that harbor a highly conserved ∼150-residue ellipsoid-shaped core UBC domain, which interacts with the E1 and the E3 ligase at different steps during the conjugation reaction. Whereas most E2 enzymes consist of this UBC core domain only, others also contain N- and/or C-terminal extensions. UBE2Z harbors both N- and C-terminal extensions on top of the UBC core domain, classifying it as a class IV E2 enzyme (20). The structure and role for such extensions have not been extensively studied. Some of these may be intrinsically disordered (22), whereas others have defined structures such as the C-terminal extension of class III E2 UBE2K (also known as E2–25K), which folds as a UBA domain (ubiquitin associated domain) and likely interacts with ubiquitin, or the N- and C-terminal extensions of class IV E2 domain of BIRC6 (baculoviral inhibitor of apoptosis repeat containing 6), whose function is as yet undefined despite the availability of structural data (PDB code 3CEG) (23).

To date the elements that direct specificity between FAT10, UBA6, and UBE2Z are not known. Here we report the crystal structure of class IV E2 enzyme UBE2Z. We also show in biochemical assays that a specific LB loop region and the N-terminal extension in UBE2Z are essential for selectivity toward UBA6 and demonstrate that the FAT10 C-terminal tail CYCI motif hinders both FAT10 activation and transfer to the E2 enzyme.

## Experimental Procedures

### 

#### 

##### Cloning and Constructs

UBE2Z variants, UbcH5c, and BIRC6 were cloned into the *pETNKI-His-3C-LIC-Kan* vector following a ligation-independent cloning procedure (24), with primers designed using ProteinCCD (25). UBA6 was amplified using primers 5′-catgccatggaaggatccgagcctgtggcc-3′ and 5′-ccgctcgagatcagtgtcatgactgaagta-3′ and cloned into the pET24d vector (Novagen) using NcoI and XhoI sites. FAT10_LRLR_ and Ub_CYCI_ mutants were generated using the QuikChange mutagenesis kit (Agilent). The *ip-SUMO-FAT10* construct used for FAT10 expression was a gift from Marcus Groettrup.

##### Protein Expression, Purification, and Labeling

Untagged ubiquitin variants and UBA1 were purified as previously described (26, 27). All other proteins used in this study were overexpressed in BL21(DE3) *Escherichia coli* cells using 0.1 mm isopropyl 1-thio-β-d-galactopyranoside induction overnight at 15 °C. Purification of proteins was performed on Talon beads (Clontech) in 50 mm HEPES (pH 7.5), 300 mm NaCl, 1 mm TCEP, and 10% (w/v) glycerol and eluted using 250 mm imidazole (pH 7.5). E2 variants were dialyzed overnight at 4 °C against 20 mm HEPES (pH 7.5), 50 mm NaCl, 1 mm TCEP, and 10% (w/v) glycerol in the presence of HRV 3C protease to remove His_6_ tags. UBA6 and E2s were further purified using a ResourceQ column (GE Healthcare) using a gradient to 1 m NaCl. Dialysis was done in the presence of SENP2 for FAT10 variants to remove the His_6_-SUMO tag, and proteins were further purified using a ResourceS column (GE Healthcare) with a gradient to 1 m NaCl. All proteins were finally purified using size exclusion chromatography on either Superdex75 or Superdex200 columns (GE Healthcare) as appropriate in a buffer containing 20 mm HEPES (pH 7.5), 150 mm NaCl, 1 mm TCEP, and 10% (w/v) glycerol. Concentrated protein aliquots were stored at −80 °C. All protein concentrations indicated correspond to total protein and are based on UV absorbance at 280 nm.

Cyanine5-NHS ester (Lumiprobe) was attached to UBLs or E2 enzymes following the directions from the manufacturer. Briefly, proteins in 20 mm HEPES (pH 7.5), 150 mm NaCl, 1 mm TCEP, and 10% (w/v) glycerol were incubated for 10 min in the presence of the fluorophore at room temperature such that the label:protein ratio would be <1. Labeled protein was then separated from free dye on PD10 desalting columns (GE Healthcare). It was verified using competition experiments between labeled and unlabeled UBLs in UBE2Z loading assays that the Cyanine5 label does not perturb kinetic measurements. Time-course experiments analyzing UBE2Z loading by UBLs also indicate that Cyanine5-labeled UBLs behave in a similar way as unlabeled UBLs.

##### Crystallization

Initial UBE2Z microcrystals were grown using protein concentrated to 12 mg·ml^−1^ in a crystallization buffer containing 25% (w/v) PEG6000 and 0.1 m Tris (pH 8.0). A seed stock was generated from these microcrystals and used in cross-seeding experiments. Diffraction quality crystals grew at room temperature in 8–25% (w/v) PEG1500, 0.1 m MMT (D-malic acid-MES-TRIS) buffer at a pH ranging between 5.0 and 7.5. Crystals were cryoprotected in mother liquor supplemented with 25% ethylene glycol before they were flash-frozen in liquid nitrogen.

##### Structure Solution and Refinement

Data were collected on beamlines ID23−1 at European Synchrotron Radiation Facility (France). UBE2Z crystals were in space group P2_1_2_1_2 and diffracted to a resolution of 2.1 Å. Data reduction was done using XDS and XSCALE (28, 29). Molecular replacement trials were performed using PHASER (30) with UBE2D3 (PDB code 1X23) as the search model for UBE2Z. The initial model produced was improved upon using phenix.mr_rosetta (31). Experimental phases were further acquired using crystals soaked in methylmercuric chloride. Iterative rounds of refinement were performed using either REFMAC (32) from the CCP4 suite (33) or BUSTER (34) and were interspersed with manual building in COOT (35). Both the x-ray weight and B-factor restraint weight in REFMAC were optimized to reduce the *R*_work_/*R*_free_ gap during refinement. Refined structures were optimized using local (36) and webserver (37) versions of PDB_REDO and were validated using the Molprobity server (38). Structure figures were generated using PyMOL (39). The UBE2Z structure has been deposited in the Protein Data Bank with accession code 5A4P.

##### Small Angle X-ray Scattering Data Acquisition and Processing

Small angle x-ray scattering measurements on UBE2Z_Nter_ (residues 1–93), prepared in buffer 20 mm HEPES (pH 7.5), 150 mm NaCl, 10% (w/v) glycerol, and 1 mm TCEP, were performed on beamline BM29 at European Synchrotron Radiation Facility (France). The samples were thawed and centrifuged at high speed for 5 min before data acquisition. Samples were exposed to x-rays in a measuring cell cooled to 4 °C. Data were processed with Primus (40) from the ATSAS software package (41). The quality of data at low angles was assessed from Guinier plots.

##### E1 and E2 Loading Assays

All loading assays were performed for 30 min at 32 °C in a buffer containing 20 mm HEPES (pH 7.5), 150 mm NaCl and 10% (w/v) glycerol. E1 and E2 loading assays were performed using 25 μm UBL, 5 μm E1, 5 μm E2 (where required), and 2.5 mm concentrations each of ATP and MgCl_2_. Reactions were stopped using non-reducing SDS-PAGE loading buffer. Samples were separated on 4–12% Bis-Tris NuPAGE gels (Invitrogen) in MES buffer or on 3–8% Tris acetate gels. Gels for E1 loading assays were visualized after Coomassie staining. E2 loading assays were performed using Cy5-labeled E2 proteins, and band detection was performed using a LAS4000 imager (GE Healthcare). The different qualitative end-point assays were performed using freshly thawed protein aliquots, and the results obtained were reproducible across at least three different protein batches.

##### Kinetic Analysis of E2∼UBL Formation

E2∼UBL designates thioester-linked covalent complexes between E2 enzymes and UBLs. E2-loading rates were calculated from a gel-based assay. E2-loading reactions were performed with 0.5 μm E1, 5 μm Cy5-labeled UBL, 2.5 mm concentrations each of MgCl_2_ and ATP, and 0–32 μm E2 in a buffer composed of 20 mm HEPES (pH 7.5), 150 mm NaCl, and 10% (w/v) glycerol, with a final reaction volume of 30 μl. Reactions were stopped with non-reducing SDS-PAGE buffer after 45 s for reactions including ubiquitin or FAT10_LRLR_ or after 4 min for reactions including FAT10 or Ub_CYCI_. Reaction times used were determined based on time-course assays measuring UBL loading on UBE2Z variants. Assay samples were separated on 4–12% NuPAGE gels (Invitrogen) in MES buffer, and E2∼UBL bands were quantified using the LAS4000 imager associated with the ImageQuantTL software (GE Healthcare). Quantification was done with data obtained within linear range of exposure. Initial rates for UBL transfer from the E1 to the E2 enzymes were determined at different E2 concentrations (1–32 μm) and were fitted using non-linear regression to the Michaelis-Menten model or to a substrate inhibition effect equation using Prism 6.05 (Graphpad Software Inc.). Experiments were performed at least three times using different protein batches. Mean values ± S.E. are plotted. Generating Ubl-loaded UBE2Z variants requires the combined activity of both UBA6 and UBE2Z enzymes. Because the Michaelis-Menten model used in our enzyme characterization assumes that a single enzyme is involved in the reaction, apparent *V*_max_ and apparent *K_m_* values are reported, reflecting the combined activity of the UBA6-UBE2Z couple. A control with the highest E2 concentration for each experimental setup showed that the E2∼UBL thioester bond was reducible in the presence of 5 mm β-mercaptoethanol.

## Results

### 

#### 

##### UBE2Z Structure: Divergence from UBC Core Domain and Emerging Similarity between Class IV E2 Enzymes

We have solved the structure of UBE2Z to a resolution of 2.1 Å (Table 1) by molecular replacement using the structure of UBE2D3 (also known as UBCH5C) (PDB code 1X23) as a model.

**TABLE 1:** **Data collection and refinement statistics** ESRF, European Synchrotron Radiation Facility.

	UBE2Z
**Data collection**	ID23-EH1 (ESRF)
Space group	P22121
Cell dimensions	
*a*, *b*, *c* (Å)	45.33, 57.81, 105.11
α, β, γ (°)	90
Resolution (Å)	38.89-2.10 (2.16-2.10)*^a^*
*R*_merge_	0.097 (0.593)
〈*I* /σ(*I*)〉	8.0 (1.6)
Completeness (%)	94.3 (95.2)
Redundancy	2.7 (2.7)

**Refinement**	
Resolution (Å)	38.88-2.10
No. reflections	16,612
*R*_work_/*R*_free_	0.23/0.27
No. atoms	
Protein	1839
Ligand/ion	49
Water	23
B-factors (Å^2^)	
Protein	57.9
Ligand/ion	80.4
Water	35.9
Root mean square deviations	
Bond lengths (Å)	0.010
Bond angles (°)	1.01
Ramachandran statistics	
Outliers (%)	0
Favored (%)	97.35

*^a^* Values in parentheses are for the highest resolution shell. The structure was solved by molecular replacement with data from a single crystal.

**FIGURE 1.**
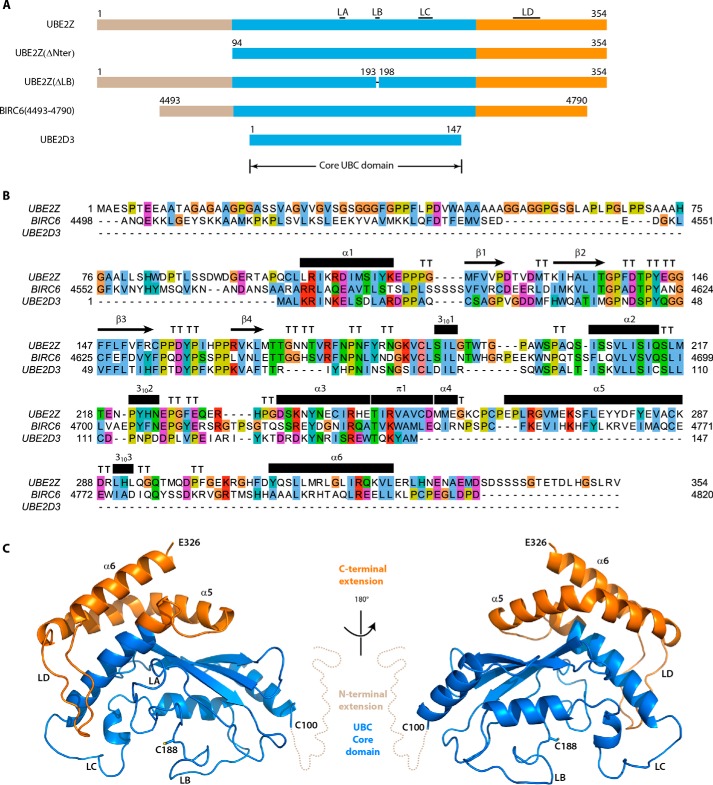
**Structural organization of UBE2Z.** *A*, schematic view of E2 enzyme constructs. The core UBC domain is colored in *blue*, N-terminal extensions are in *silicon*, and C-terminal extensions are in *orange*. Construct boundaries as well as the positions for loops LA, LB, LC, and LD are indicated. *B*, sequence alignment between UBE2Z, BIRC6 and UBE2D3. Residues are colored according to the ClustalX coloring scheme. The secondary structure of UBE2Z, calculated using DSSP, is indicated *above the alignment. C*, schematic view of the structure of UBE2Z with a similar color scheme as in *A*. Two orientations are presented corresponding to a 180° rotation around the *y* axis. The N-terminal extension (residues 1–99) is not visible in this structure and is represented as a *dotted line*. The catalytic Cys-188 residue is represented as *sticks*.

UBE2Z is a 354-residue-long atypical ubiquitin conjugating enzyme comprising ∼100-residue long N- and C-terminal extensions on top of the conserved core UBC domain (Fig. 1, *A–C*), classifying it as a class IV E2 enzyme. The core domain of UBE2Z shares ∼34% sequence identity with UBE2D3 (Fig. 1*B*).

**FIGURE 2.**
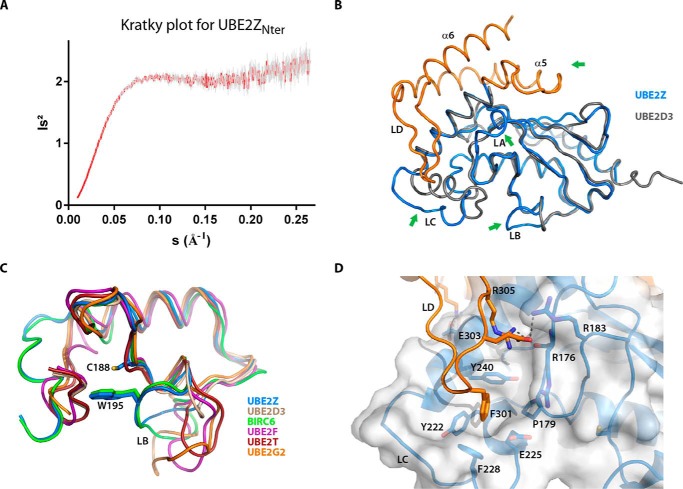
**Analysis of UBE2Z loop regions and N-terminal extension.** *A*, Kratky plot (*Is^2^* v/s *s*) for small angle x-ray scattering data acquired on UBE2Z_Nter_ (residues 1–93) indicating that the protein is unfolded. Data points are in *red*, and *error bars* are in *gray. B*, superposition of UBE2D3 lacking a C-terminal extension (*gray*) on UBE2Z (*colored blue* for its core UBC domain and *orange* for its C-terminal extension). Divergence between the structures for the two proteins is indicated by *green arrows. C*, close-up schematic view of the UBE2Z LB loop region (*blue*), with the catalytic Cys-188 and LB loop Trp-195 shown as *sticks*. The superposition of E2 enzymes UBE2D3, BIRC6, UBE2F, UBE2T, and UBE2G2 (PDB codes 1X23, 3CEG, 3FN1, 4CCG, and 2CYX, respectively) on UBE2Z is also represented. BIRC6 Trp-4645 corresponding to UBE2Z Trp-195 is shown as *sticks. D*, schematic representation for the interaction between UBE2Z C-terminal extension loop LD (*orange*) and the UBE2Z core UBC domain (*blue*). The surface of the UBC domain is represented in *transparency*, and residues involved in the interaction with tip of loop LD are shown as *sticks*.

Whereas full-length UBE2Z was used in our crystallization trials, our structure lacks the N-terminal 98 residues of the protein corresponding to the UBE2Z N-terminal extension. This region showed poor sequence similarity with class II or class IV E2 enzymes that contain N-terminal extensions (Fig. 1*B*) but is well conserved in mammalian UBE2Z orthologs (15). The amino acid composition of the UBE2Z N-terminal extension was highly biased toward alanine (23%), glycine (20%), proline (12%), and serine (10%) residues (Fig. 1*B*), suggesting that this region in UBE2Z is likely to be intrinsically disordered (42). Kratky plots based on small angle x-ray scattering data acquired on this N-terminal UBE2Z region (residues 1–93) indeed suggest that the N-terminal extension does not acquire a globular fold but is most likely disordered (Fig. 2*A*). Residues 327–354 at the C terminus of UBE2Z could also not be built in our structure and are predicted to be unstructured. The rest of our structure (residues 99–326) is well defined, revealing the UBC core domain and the C-terminal extension of UBE2Z (Figs. 1*C* and 2*B*).

The UBE2Z core domain adopts the characteristic ellipsoid shape of UBC domains (Fig. 1*C*) but also harbors two extensions termed loops LA (residues 169–173) and LB (residues 194–197) compared with the prototypical class I E2 enzyme UBE2D3 (Fig. 2*B*). Interestingly, UBE2Z loop LA is absent in all other E2 enzymes except for class IV E2 BIRC6, and UBE2Z constructs lacking this region yielded insoluble protein. On the other hand, loop regions equivalent to the UBE2Z LB exist in other E2 enzymes such as UBE2T, UBE2G2, or UBE2F, where they adopt different sizes and offer different degrees of closure over the catalytic cysteine of the conjugating enzymes (PDB codes 4CCG, 2CYX, and 3FN1, respectively) (Fig. 2*C*). Contrary to those E2 enzymes, UBE2Z and BIRC6 (PDB code 3CEG) display similarly oriented LB loops, which harbor a conserved tryptophan residue (Trp-195 in UBE2Z) that contributes largely in masking the catalytic cysteines in these enzymes (Fig. 2*C*). The UBE2Z catalytic cysteine indeed displays an accessible surface area of 1.9 Å^2^ as opposed to 17.2 Å^2^ for the catalytic cysteine in UBE2D3 (Fig. 2*C*), which lacks an insert corresponding to UBE2Z loop LB. UBE2Z loop LB residues are well conserved among other class IV E2s UBE2O and BIRC6. The UBE2Z core domain structure further deviates from that of the UBC domain as it lacks the third UBC helix, which is replaced here by a loop LC (Figs. 1*C* and 2*B*).

The UBE2Z C-terminal extension is packed against the backside of the core UBC domain similar to BIRC6 and comprises two helices (α5 and α6), which enclose a loop region (loop LD) (Figs. 1*C* and 2*D*). This extension shares a large interface with the core domain (1467 Å^2^) and is stabilized by eight hydrogen bonds and six salt bridges. The UBE2Z LD loop comprises 18 residues as opposed to the shorter 5-residue-long BIRC6 loop. It interacts with the UBE2Z core domain LC loop through Phe-301 located at its tip. Phe-301 is nested in a hydrophobic crevice made up of residues Arg-176, Pro-179, Tyr-222, Glu-225, Phe-228, and Tyr-240 originating from the E2 core domain (Fig. 2*D*). Despite the similarity in the structural organization between the UBE2Z and BIRC6 C-terminal extensions, there is poor sequence conservation between these protein regions (15.5% identity) (Fig. 1*B*).

##### UBE2Z N-terminal Extension and Loop LB Are Essential for Selectivity toward UBA6

We generated a series of UBE2Z constructs to test the role of the extensions in UBE2Z. Although we did not achieve soluble protein when we removed the C terminus or the LA loop, we were able to obtain purified UBE2Z lacking the N-terminal domain or the LB loop. These UBE2Z variants were used to investigate how different regions in UBE2Z are involved both in the specific recognition between the E2 and the UBL-loaded E1 enzyme and in UBL transfer from the E1 to the E2 enzyme. Our assays compared the activities to UBE2D3, which contains only the core UBC domain, and class IV E2 enzyme BIRC6_4493–4790_ (including residues 4493–4790, later called BIRC6), which comprises extensions as well as the LA and LB inserts similar to UBE2Z.

**FIGURE 3.**
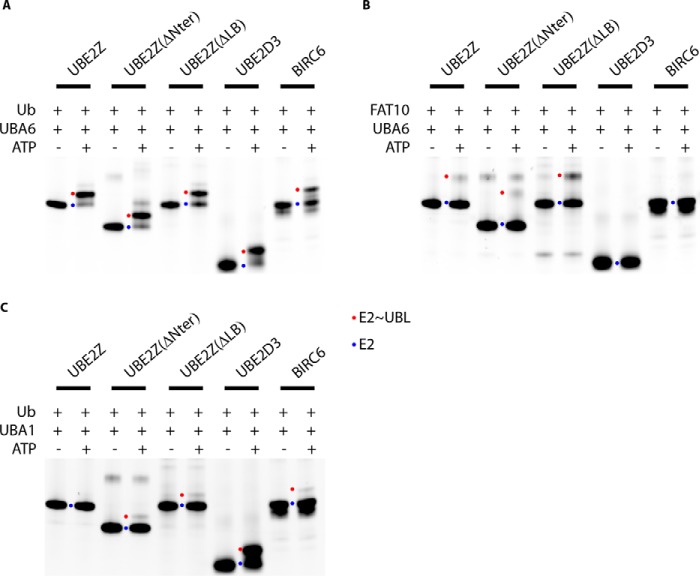
**Specificity in UBL charging on E2 variants.** E2-loading assays are represented using UBA6 and ubiquitin (*A*) or FAT10 (*B*) and using UBA1 and ubiquitin (*C*). Each assay was performed without or with ATP, and uncharged E2s are indicated with a *blue dot*, whereas UBL-loaded E2s are indicated with a *red dot*. Cy5-labeled E2 variants were used in this assay and specifically allow visualization of the unloaded and loaded E2s under non-reducing conditions.

Our *in vitro* E2 loading assays show that ubiquitin (Fig. 3*A*) or FAT10 loading (Fig. 3*B*) onto UBE2Z_ΔNter_ or onto UBE2Z_ΔLB_ is not abolished, suggesting that in our reaction conditions the N-terminal extension or the LB loop region is not essential in UBA6∼UBL recognition or in the transfer of the UBL to the E2 enzyme. As expected, our UBE2Z variants are the only E2s in our assays capable of accepting FAT10, but we nevertheless find that UBA6 charges UBE2Z with FAT10 less efficiently than with ubiquitin.

Interestingly, despite sharing common structural features with UBE2Z such as a similarly organized C-terminal extension or LB loop, BIRC6 is non-functional in FAT10 loading in the same way as UBE2D3 (Fig. 3*B*). However, we find that BIRC6 seems to be loaded with ubiquitin more efficiently by UBA6 (Fig. 3*A*) than by UBA1 (Fig. 3*C*).

It has previously been shown that UBE2Z is a selective E2 enzyme, functioning in ubiquitination only with UBA6 (15). However, both UBE2Z_ΔLB_ and UBE2Z_ΔNter_ can be loaded with ubiquitin by UBA6 (Fig. 3*A*) but also by UBA1, albeit with much lower efficiency than observed for UBE2D3 (Fig. 3*C*). These UBE2Z variants, therefore, lose the ability to discriminate against ubiquitin-loaded UBA1 (Fig. 3*C*), suggesting that both the N-terminal extension and the loop LB regions are required for UBE2Z to maintain its selectivity toward UBA6.

##### UBE2Z N-terminal Extension and LB Loop Contribute Differently to Ubiquitin Loading Compared with FAT10 Loading

We then investigated the kinetics of UBL loading on UBE2Z variants using E2-UBL thioester formation assays. UBA6 was used to load either ubiquitin or FAT10 on our UBE2Z variants, and initial rate data were generated to derive apparent *V*_max_ and *K_m_* values.

**FIGURE 4.**
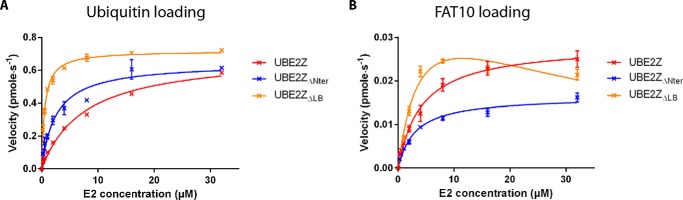
**Enzyme kinetics for UBL loading on UBE2Z variants.** Fits of kinetic data for E2-thioester formation assays are shown for UBE2Z (*red*), UBE2Z_ΔNter_ (*blue*), and UBE2Z_ΔLB_ (*orange*) variants using ubiquitin (*A*) or FAT10 (*B*) and UBA6 as the E1 enzyme.

Our data, fitted to the Michaelis-Menten model, show that UBE2Z, UBE2Z_ΔLB_, and UBE2Z_ΔNter_ display similar catalytic rates during ubiquitin loading with apparent *V*_max_ values between 0.6 and 0.7 pmol·s^−1^ (Fig. 4*A* and Table 2). However, apparent *K_m_* values for ubiquitin loading decrease drastically when the UBE2Z N-terminal extension or loop LB regions are absent (∼3- and ∼15-fold, respectively), suggesting that these regions hinder UBE2Z binding to ubiquitin-loaded UBA6 (Fig. 4*A* and Table 2).

**TABLE 2:** **Kinetic parameters for loading of Ubiquitin or FAT10 variants on UBE2Z variants.**

UBL	E2 variant	Apparent *V*_max_	Apparent *K_m_*
		*pmol*·*s*^−*1*^	μ*m*
Ubiquitin	UBE2Z	0.70 ± 0.02	7.56 ± 0.65
Ubiquitin	UBE2Z_ΔNter_	0.65 ± 0.03	2.49 ± 0.46
Ubiquitin	UBE2Z_ΔLB_	0.72 ± 0.01	0.53 ± 0.04
FAT10	UBE2Z	0.03 ± 0.00	4.49 ± 0.79
FAT10	UBE2Z_ΔNter_	0.02 ± 0.00	3.39 ± 0.47
FAT10	UBE2Z_ΔLB_	0.04 ± 0.01	4.15 ± 0.95
FAT10_LRLR_	UBE2Z	0.72 ± 0.01	8.60 ± 0.33
Ub_CYCI_	UBE2Z	0.04 ± 0.00	2.92 ± 0.49

Analyzing FAT10 loading on UBE2Z variants shows that UBE2Z_ΔLB_ behaves differently from UBE2Z and UBE2Z_ΔNter_ (Fig. 4*B*). Whereas UBE2Z and UBE2Z_ΔNter_ data were fitted to the Michaelis-Menten model, UBE2Z_ΔLB_ data could be fitted with a substrate inhibition effect as previously observed for UBE2D2 (also known as UBC5b) (43) or UBE2B (also known as UBC2B) (44) loading with ubiquitin by UBA1. This inhibition effect likely results from non-productive binding of UBE2Z_ΔLB_ to FAT10-loaded UBA6. Little variation was observed in apparent *K_m_* (3.4–4.9 μm) for our UBE2Z variants during FAT10 loading (Fig. 4*B* and Table 2), contrary to what was observed during ubiquitin charging on the E2 constructs. This indicates that the N-terminal extension or the LB loop does not adversely affect UBE2Z interaction with FAT10-loaded UBA6. Instead, variations are visible for apparent *V*_max_ values recorded (0.02–0.04 pmol·s^−1^) for E2 loading by FAT10, indicating that these regions possibly affect the rate of transfer.

Our data correlate with previous kinetic measurements of UBE2Z loading by ubiquitin or FAT10 in the presence of UBA6 (45). Indeed, both studies show that ubiquitin is loaded more efficiently than FAT10 on UBE2Z, albeit with different absolute *K*_cat_ values, likely reflecting differences in reaction conditions.

##### FAT10 CYCI C-terminal Peptide Constitutes a Major Selectivity Marker toward Both UBA6 and UBE2Z

**FIGURE 5.**
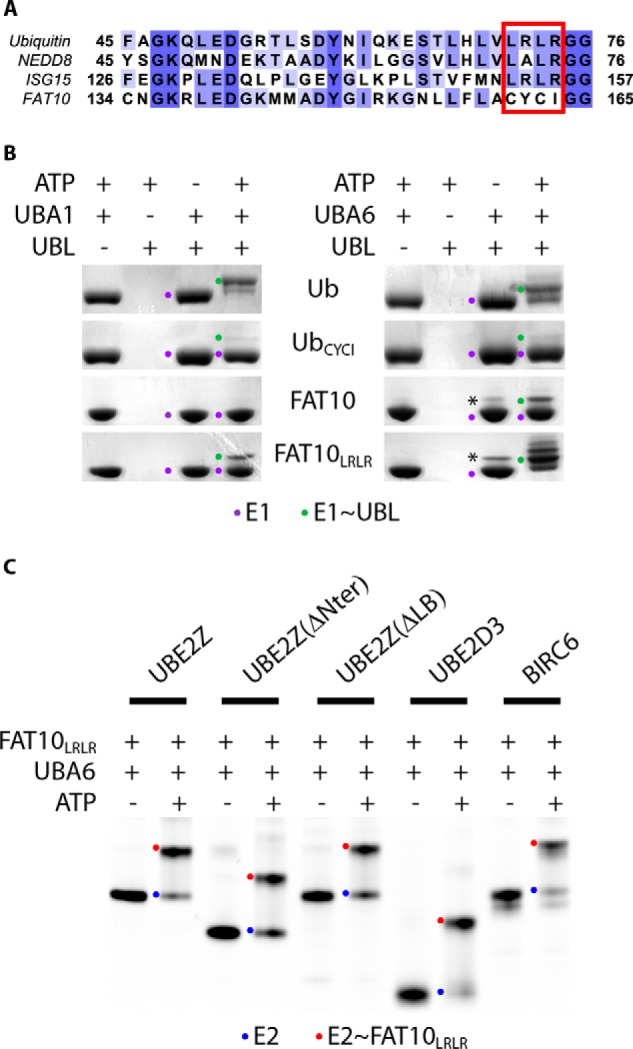
**Specificity toward E1 and E2 enzymes lies in the UBL C-terminal tail.** *A*, sequence alignment between the C-terminal residues of ubiquitin, NEDD8, ISG15, and FAT10. Sequence limits are indicated for each protein. The C-terminal LXLR motif is highlighted by a *red rectangle*. Residues are colored according to conservation, with *dark blue*-colored residues highly conserved. *B*, Coomassie stained gel showing E1 charging of UBA1 (*left*) and UBA6 (*right*) by different UBL variants as indicated under non-reducing conditions. *Asterisks* (*) indicate nonspecific bands likely corresponding to disulfide-bridged E1-UBL complexes. Unloaded E1s are indicated with a *purple dot*, whereas UBL-loaded E1s are indicated with a *green dot*. Bands above those corresponding to the UBL-loaded E1s likely correspond to the auto-ubiquitinated or auto-fatylated species of the E1 enzyme as applicable. *C*, E2 loading assays using FAT10_LRLR_ and UBA6 on different E2 variants. Cy5-labeled E2 variants were used in this assay and specifically allow visualization of the unloaded (*blue dots*) and loaded E2s (*red dots*) under non-reducing conditions.

Among different UBLs, ubiquitin, ISG15, and NEDD8 share a similar C-terminal L*X*LR tetrapeptide (where L is a leucine, *X* is an alanine or an arginine, and *R* is an arginine) located before the conserved diglycine motif (Fig. 5*A*). Previous studies have shown that the presence of an arginine at position 72 in ubiquitin prevents its activation by the NEDD8 E1 enzyme (46, 47) and that this tetrapeptide motif in UBLs contributes to specificity for their cognate E1 enzyme (46–48). We, therefore, investigated whether the FAT10 CYCI motif (where C is a cysteine, Y is a tyrosine, and I is an isoleucine) could hold such a role and switched the FAT10 CYCI motif to the ubiquitin LRLR to generate the FAT10_LRLR_ mutant. We used our different UBL variants to perform loading assays on UBA6 or UBA1. As expected, we find that ubiquitin is activated by both UBA1 and UBA6 (Fig. 5*B*), whereas FAT10 can be loaded on UBA6 but not on UBA1 (Fig. 5*B*). Furthermore, our data clearly show that switching the FAT10 CYCI to an LRLR motif, as found in ubiquitin, leads to a loss in E1 selectivity as FAT10_LRLR_ can be loaded on UBA1 contrary to wild type FAT10 (Fig. 5*B*). We further generated Ub_CYCI_, a ubiquitin mutant incorporating the FAT10 CYCI motif instead of the LRLR residues. As expected, Ub_CYCI_ can still be loaded on UBA6 but nevertheless retained its ability to be loaded onto UBA1, albeit with markedly lower efficiency (Fig. 5*B*). Altogether, these data suggest that the CYCI motif is required for FAT10 selectivity toward UBA6 but also that other as yet unidentified features in this UBL may prevent its activation by UBA1. Interestingly, both E1 enzymes can activate Ub_CYCI_ but with lower efficiency than was observed with ubiquitin (Fig. 5*B*), indicating that the CYCI motif negatively impacts E1 charging compared with the LRLR tetrapeptide.

Comparing E2 loading by FAT10 (Fig. 3*B*) or FAT10_LRLR_ (Fig. 5*C*) in the presence of UBA6 clearly showed that FAT10_LRLR_ but not FAT10 can be loaded onto E2s other than UBE2Z variants, such as UBE2D3 or BIRC6. Our data, therefore, reveal that the FAT10 CYCI motif plays an important role in FAT10 selectivity for UBE2Z.

##### C-terminal CYCI Peptide in FAT10 Limits Transfer Rates onto UBE2Z

**FIGURE 6.**
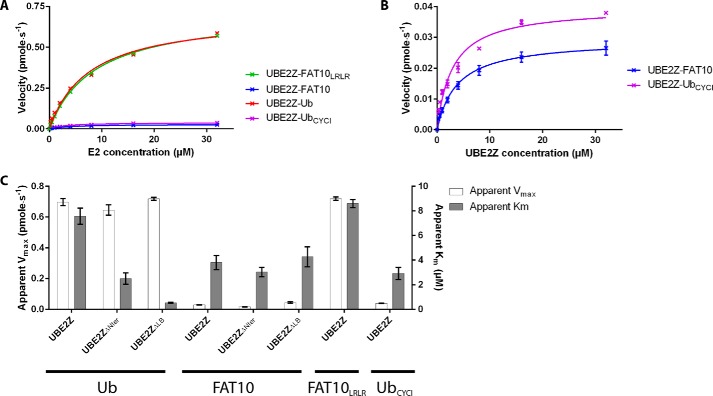
**Enzyme kinetics for loading of UBL variants on UBE2Z.** *A*, Michaelis-Menten fits for E2-thioester formation assays are shown for ubiquitin (*red*), FAT10 (*blue*), and Ub_CYCI_ (*purple*) on UBE2Z and for FAT10_LRLR_ and on UBE2Z (*green*). *B*, the expanded view of fits for FAT10 and Ub_CYCI_ loading onto UBE2Z are shown in the same colors as previously. *C*, histogram summarizing kinetic parameters apparent *K_m_* and apparent *V*_max_ for the loading of different UBL and UBL mutants onto UBE2Z variants.

Comparing the transfer of ubiquitin to that of FAT10 onto UBE2Z showed only a minor difference in apparent *K_m_* (1.7 fold) but a striking difference in apparent *V*_max_ (∼25-fold) (Fig. 6, *A*–C, and Table 2). Such a large variation cannot be explained by the small difference previously shown between activation rates of ubiquitin and FAT10 by UBA6 (45). We tested whether the LRLR *versus* CYCI sequence before the diglycine motif at the C-terminal end of the UBLs could explain these differences (Fig. 5*A*). We quantified transfer rates for the FAT10_LRLR_ mutant onto UBE2Z using UBA6 and found apparent *K_m_* and apparent *V*_max_ values comparable with those observed with ubiquitin (8.7 μm and 0.7 pmol·s^−1^, respectively) (Fig. 6, *A* and *C*, and Table 2).

We then investigated how the CYCI tetrapeptide affects Ub_CYCI_ transfer onto UBE2Z and found that it is transferred onto UBE2Z at a lower rate, comparable with that of FAT10 (Fig. 6, *A* and *C*, and Table 2). This lower transfer rate observed when the UBL harbors a C-terminal CYCI tetrapeptide motif strongly suggests that the catalysis of the trans-thioesterification reaction is suboptimal in the presence of this CYCI motif. Overall, our data indicate that the CYCI peptide in FAT10 lowers the UBL transfer rate from the E1 to the E2 enzyme and hence contributes to selectivity toward UBE2Z.

## Discussion

The FAT10 conjugation pathway involves an intricate interplay of specific protein-protein interactions that define both substrate and enzyme selectivity. Whereas FAT10 has been described to function only with UBA6 as its sole activating enzyme (14) and with UBE2Z as its conjugating enzyme (16), these E1 and E2 enzymes are also involved in ubiquitination reactions (14, 15). On the other hand, UBE2Z has been shown to function with UBA6 but not with UBA1 (15). Previous studies indicate that the E1 ubiquitin-fold domain is involved in E2 enzyme recruitment onto the UBL-loaded E1 enzyme (49, 50), but UFD swapping experiments between UBA6 and UBA1 were not sufficient to alter UBE2Z specificity toward UBA6 (15). This suggests that other structural elements from the E1 or E2 enzymes generate specific interactions between UBE2Z and UBA6.

Through this work we defined how specificity is achieved among the different partners in the FAT10 conjugation pathway. We found that FAT10 selectivity toward UBA6 resides in the CYCI tetrapeptide preceding the C-terminal diglycine motif conserved in all UBLs. Previous work has shown that this region in UBLs is important in providing selectivity toward their cognate E1 enzymes. Indeed, residue Arg-72 in ubiquitin prevents its adenylation by the heterodimeric NEDD8 activating enzyme, whereas an alanine at the same position allows activation by the same E1 (51). Furthermore, ubiquitin variants harboring mutations at their C-terminal end are activated with lower efficiency than wild type ubiquitin by UBA1 (48). Interestingly, we show that this same CYCI tetrapeptide is responsible for FAT10 selectivity toward UBE2Z. Previous studies suggest that UBL selectivity toward E2s is achieved through specific recruitment of the cognate E2 by the E1 UFD (49, 50, 52). In the case of UBE2Z, it has been shown that UBA6 cannot proceed with ubiquitin transfer onto the E2 enzyme in the absence of its cognate UFD (15). However, UBA6, being itself bi-specific for FAT10 and ubiquitin, can interact with UBE2Z and also with other E2 enzymes involved only in ubiquitination such as UBE2D3, suggesting that the UBA6 UFD does not harbor all of the selectivity determinants geared toward FAT10 conjugation. Previous studies show that UBE2M (also known as UBC12) is recruited to the NEDD8 E1 enzyme through specific recognition between the E1 UFD and the E2 core UBC domain N-terminal helix (50) but that a specific sequence in the E2 enzyme prevents interaction with UBA1 (53). We similarly show that the LB loop and N-terminal extension in UBE2Z are essential in preventing the interaction between the E2 enzyme and UBA1. This LB loop region is well conserved among class IV E2 enzymes BIRC6 and UBE2O and might also direct their selectivity toward UBA6. Its position close to the E2 catalytic cysteine could explain its influence on the rate of UBL transfer from the E1 to the E2 enzyme as observed in our FAT10 loading assays on UBE2Z. On the other hand, the N-terminal extension in UBE2Z is not conserved in other E2 enzymes and, unlike the corresponding domain in BIRC6, is not structured. The disordered nature of this extension likely results from its compositional bias, with stretches of Gly/Ser-rich regions. We believe that the UBE2Z N-terminal extension might be involved in specific interactions with UBA6 similar to what is observed between the unstructured UBE2M N-terminal extension and the NEDD8 E1 (19). This interaction could contribute to orienting UBE2Z such that the transfer of CYCI motif containing FAT10 is favored as opposed to LRLR tetrapeptide containing ubiquitin or FAT10_LRLR_.

Our structural data show that despite low sequence similarity between C-terminal extensions of class IV E2 enzymes UBE2Z and BIRC6, these adopt a similar double helical arrangement, stacked on the “backside” of the E2 core UBC domain. Lack of structural data on UBE2O, the other member of this class of E2s, prevents us from generalizing on a conserved structural organization of C-terminal extensions in this family of E2 enzymes, but nevertheless, secondary structure predictions suggest that the UBE2O C-terminal extension could also comprise two helices enclosing a large loop region.

Interestingly, we find that the CYCI motif in FAT10 hinders both FAT10 activation by the E1 enzyme and its charging on E2 enzymes. Contrary to UBA6, UBA1 is unable to activate CYCI motif-containing FAT10, whereas both enzymes perform badly with Ub_CYCI_ activation. It has previously been shown that FAT10 binds non-covalently to UBA6 in the absence of ATP (45), and it is likely that this interaction enhances the activation of FAT10 by UBA6 despite the presence of a CYCI tetrapeptide in this UBL. Lower thioester formation rates on UBE2Z are also observed with FAT10 compared with our FAT10_LRLR_ mutant. Similarly, we find that Ub_CYCI_ is loaded less efficiently onto UBE2Z when compared with ubiquitin, suggesting that catalysis of the trans-thioesterification reaction in the presence of the CYCI motif is not favored as opposed to UBLs harboring an LRLR motif. We also show that FAT10 can only be transferred onto UBE2Z variants, whereas FAT10_LRLR_ can also be loaded onto other E2s.

Intriguingly, despite being the only E2 enzyme involved in FAT10 conjugation, UBE2Z is only poorly charged with FAT10 in our *in vitro* assays when compared with ubiquitin. The low transfer rates we observe suggest that other unidentified partners might be required to improve FAT10 loading onto UBE2Z. This also reflects the tight level of regulation involved in the FAT10 conjugation pathway. We believe that the CYCI motif in FAT10 acts as a limiting factor for both activation by UBA6 and transfer onto UBE2Z and that specificity is achieved through targeted interactions between FAT10 and these enzymes.

## Author Contributions

P. R. designed, supervised, and performed the experiments and interpreted the data. T. K. S. designed and supervised the experiments. J. S. designed and performed the experiments and interpreted the data. D. M. and F. D. provided technical assistance. All authors contributed to writing of the manuscript and reviewed and approved the final version of the manuscript.

## Footnotes

2 The abbreviations used are:

UBL: ubiquitin-like modifier
UFD: ubiquitin fold domain
UBC: ubiquitin conjugating enzyme
TCEP: tris(2-carboxyethyl)phosphine
Bis-Tris: 2-[bis(2-hydroxyethyl)amino]-2-(hydroxymethyl)propane-1,3-diol
Ub: ubiquitin.
